# Audit of country clients accessing metropolitan podiatry services in South Australia

**DOI:** 10.1186/1757-1146-4-S1-P60

**Published:** 2011-05-20

**Authors:** Noami Zakarias

**Affiliations:** 1 Country Health SA, South Australia, 5000, Australia

## Background

The purpose of the audit was to take a snapshot in time (8 week period) of the number of people from Country Health SA (CHSA) receiving podiatry services from the Adelaide Health Service (AHS), and to trace their usage of local services. The audit also aimed to identify strategies to reduce travel and improve patient journey.

## Methods

The audit period was for 8 weeks (15/2/10-13/4/10). An OASIS (Online Analytical Statistical Information System) data report of people with CHSA postcodes using AHS services with the following search terms was generated: foot, leg, ulcer, wound, podiatry. The data report was sent to CHSA and AHS podiatry departments for verification and cross referenced against the CHSA client database.

## Results

In the 8 week audit period there were 232 occasions of service for 130 people from CHSA postcodes accessing AHS podiatry services. The main clinical presentation of people from Country Health SA accessing the Adelaide Health Service is diabetic foot complications (e.g. ulceration, amputation). Approximately half of the people accessing services (51%) were registered CHSA clients, and only a small proportion (13%) were both registered CHSA clients and CHSA podiatry clients. The other half of people were not registered CHSA clients (49%), a small proportion (14%) were previously registered CHSA clients. Of the clients that were registered CHSA clients but not CHSA podiatry clients, two-thirds of the clients accessed community nursing. Diabetes education and dietetics were also accessed by this client group. Only 8% of people from CHSA postcodes accessing AHS podiatry services were referred directly from CHSA podiatry. The remaining 92% of referrals came from medical specialists, GPs, CHSA community nursing, private podiatry and inpatient admissions. Of the 232 AHS podiatry appointments for 130 CHSA postcode clients, the majority (71%) had 1-3 appointments in the 8 week audit period. The remaining 29% of clients had 4 or more appointments in an 8 week period, resulting in fortnightly to weekly follow-up appointments at AHS podiatry clinics. Of the clients accessing AHS podiatry, the majority of clients are from inner CHSA locations. The top 4 CHSA locations for AHS podiatry service usage also had the highest podiatrist to population ratio.

## Conclusions

As the majority of the 130 people are ‘high risk’, to have only 51% as registered clients of CHSA and only 13% as registered podiatry clients is concerning as these complex clients generally require services from multiple health professionals at a high frequency, which isn’t available in most rural General Practices. With 38% of people registered CHSA clients but not podiatry clients, it is reasonable to surmise there is potential to improve local service coordination or cross-referrals to Podiatry in some areas, and/or there is lack of podiatry services or long podiatry waiting lists. 14% of people that were not current registered CHSA clients but had previously been registered with CHSA, may indicate a need for more rigorous recall systems for the ‘high risk’ clients. A recall system may have allowed for problems to be managed sooner and locally, reducing the amount of AHS podiatry presentations. The 29% of appointments that were fortnightly or more frequently are potential opportunities to reduce AHS podiatry appointments services through building staff numbers in CHSA podiatry services (where indicated), improving communication between CHSA and AHS podiatry services, and other mechanisms to improve capacity of CHSA Podiatry services such as training and equipment. Multiple strategies have been put into place since the audit period, and an audit of the same period in 2011 will be conducted as a comparison.

